# Scanning ion conductance microscopy: a nanotechnology for biological studies in live cells

**DOI:** 10.3389/fphys.2012.00483

**Published:** 2013-01-14

**Authors:** Bing-Chen Liu, Xiao-Yu Lu, Xiang Song, Ke-Yu Lei, Abdel A. Alli, Hui-Fang Bao, Douglas C. Eaton, He-Ping Ma

**Affiliations:** Department of Physiology, Emory University School of MedicineAtlanta, GA, USA

**Keywords:** live cell imaging, nanoscale topography, microvilli, cilia, endocytic pits, tight junctions, confocal microscopy, patch-clamp techniques

## Abstract

Scanning ion-conductance microscope (SICM), which enables high-resolution imaging of cell surface topography, has been developed for over two decades. However, only recently, a unique scanning mode is increasingly used in biological studies to allow SICM to detect the surface of live cells. More recently, in combination with confocal microscopy and patch-clamp electrophysiological techniques, SICM allows investigators to localize proteins or ion channels in a specific nanostructure at the cell surface. This article will briefly review SICM nanotechnique and summarize the role of SICM in biological studies.

## History

The scanning ion-conductance microscope (SICM) was invented by Hansma and co-workers at the University of California in 1989 (Hansma et al., 1989). In a wide family of scanning probe microscopes, SICM is the most recently developed one which is specifically designed for the scanning of soft, non-conductive materials bathed in electrolyte solution at sub-micrometer resolution. But for a long time, this technique was limited to imaging of flat polymer films. In 1997, Korchev et al. made significant improvements to SICM to allow the imaging of live cells without making direct contact with the sample surface (Korchev et al., 1997). A couple of years later, the same group, for the first time, mapped single active ion channels in intact cell membranes (Korchev et al., 2000b), and also developed a hybrid scanning ion conductance which allows high-resolution characterization of the cell surface and the simultaneous recording of topographic and optical images (Korchev et al., 2000c). In 2002, there were several significant breakthroughs in SICM methods. First, Gu et al. used SICM to resolve the topographical details of a living cell surface to guide patch-clamp recording of ion channel activity at a specific area of the cell surface (Gu et al., 2002). Second, Bruckbauer et al. used a nanopipette to locally and controllably deposit complex biomolecules such as DNA and protein molecules onto a surface to study these molecules at submicrometer scale (Bruckbauer et al., 2002, 2003). Third, Gorelik et al. combined SICM with confocal microscopy to perform simultaneous topographical and fluorescence imaging of cell surface (Gorelik et al., 2002). In 2003, they also used SICM to directly observe the assembly of microvillar structures in various living epithelial and nonepithelial cells (Gorelik et al., 2003). Another milestone improvement of SICM is the development of a unique way to scan the cell surface (Happel and Dietzel, 2009; Novak et al., 2009). This mode, which is called the hopping mode, is especially useful for non-contact imaging of the cell surface with a resolution better than 20 nm (Novak et al., 2009; Klenerman et al., 2011). Since then, the ability of SICM to produce high resolution images of the surface of live cells has been increasingly recognized (Yang et al., 2011, 2012; Pellegrino et al., 2012; Shevchuk et al., 2012).

## Principle

**Figure 1:**
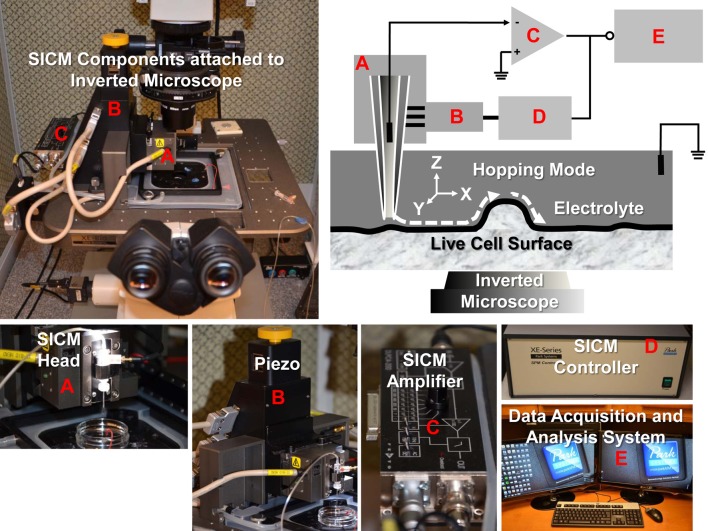
**SICM components and schematic diagram of SICM topography imaging of live cells with hopping mode.** The XE-Bio SICM setup (Park Systems Corporation) consists of SICM head **(A)**, piezo **(B)**, SICM amplifier **(C)**, SICM controller **(D)**, and data acquisition and analysis system **(E)**. Components **(A, B**, and **C)** are attached to a Nikon Eclipse T*i* inverted microscope.

The SICM consists of a glass micropipette probe, a micromanipulator, an amplifier, and an inverted microscope. The micropipette has a small opening diameter (50–100 nm) and is filled with electrolyte and lowered into a bath of electrolyte down close to the sample surface (Figure 1). A voltage is applied across the electrolyte via two electrodes; one inside the pipette and the other in the bath. As the tip of the micropipette approaches the sample surface, the ion conductance reduces because the gap that ions can flow through is decreased. Changes in the current are measured by the amplifier, and are used as a feedback signal by the scanner control unit to keep the distance between pipette tip and sample constant by applying appropriate voltages to the *Z*-piezo drive during the scanning. Therefore, the path of the tip follows the topography of the surface so that the sample topography can be recorded. There are several operating modes for SICM (addressed below).

### Direct current (DC) mode

In the DC mode, a continuous feedback mechanism moves the pipette up and down while scanning the sample to keep the pipette always in the proximity of the sample surface. Thus, a pipette-sample separation, typically equal to the pipette's inner radius, is maintained during imaging. This method suffers from poor sensitivity and resolution. Moreover, in principle, the probe does not come into contact with the surface. However, since the dependence of the ionic current on the distance between the tip and the sample is not very steep, the tip often contacts the surface of a rough sample during routine imaging. Therefore, the DC mode is now generally used only for sample with vertical protrusions below a few hundred nanometers.

### Alternating current (AC) mode

In the AC mode, a lock-in amplifier is used to detect the changes in the AC current amplitude, which is then used to control the feedback circuitry to modulate the scanning tip distance from the sample surface. This mode is much more sensitive than the DC mode, and allows the probe to be operated only a few nanometers from the sample surface. Moreover, the AC ionic current can be recorded simultaneously during the scan. As described in a previous report (Pastre et al., 2001), it can provide additional information about the local ionic environment. However, it is impossible to scan highly convoluted surfaces using either the DC or the AC mode because the SICM probe only senses locally at the tip. In other words, in these two types of mode, the side of the probe may touch the sample before the tip has sensed the presence of a very bumpy surface. Therefore, like the DC mode, the AC mode is also only suitable for samples with a relative smooth surface.

### Hopping mode/approach-retract scanning (ARS) mode

The hopping mode is also called a backstep or ARS mode. In the hopping/ARS mode, ion current is recorded while the pipette is moved vertically and repeatedly approaches and retracts from the sample surface. This new mode type no longer uses continuous feedback and raster scanning pattern. The nanopipette approaches the surface to measure the height only at selected imaging points and rapidly retracts back to a safe distance before moving laterally onto the next imaging point. The pipette approaches until the current is reduced by a predefined threshold. Typically, the pipette is still at a distance of about one inner pipette radius from the surface, usually 25–50 nm. Therefore, the hopping mode is especially powerful for imaging samples with steep slopes and is capable of imaging surfaces with rough vertical protrusions of several micrometers.

## Role in biological studies

High-resolution images of biological samples can be obtained by electron microscopy. However, the samples must be fixed before performing electron microscopy experiments. In contrast, SICM is able to reveal the morphology and dynamics of live cells at nanometer scale and to complement confocal microscopy and patch-clamp techniques. SICM can also monitor cell volume and movements, deliver mechanical and chemical stimulations to cells or cellular nanostructures, and even guide cell growth.

### Study specialized membrane structures such as microvilli, cilia, endocytic pits, and tight junctions

The apical membranes of some epithelial cells have specialized structures such as microvilli, cilia, endocytic pits, and tight junctions. SICM can provide high resolution images of these structures in live cells. In addition to revealing nanometer-scale morphology, SICM is also a powerful tool to analyze the dynamics of nanostructures of live cells. The dynamics of fine structures in cell membranes is vital for investigating cell function and facilitating the study of important physiological processes. Formerly, these structures could only be observed by scanning electron microscopy or to a lesser extent by atomic force microscopy, but both methods are only suitable for fixed samples which prevent observing the fine structural and functional changes of membranes in live cells. SICM, on the other hand, is suitable for imaging live cell surfaces bathed in electrolytes. Real-time observations of changes in the cell surface can be continuously conducted under normal physiological conditions. SICM has been used to observe directly the assembly of microvillar structures in living renal epithelial cells. The real-time nanoscale topographical images show that multiple microvilli can either form ridges or break into small isolated structures. The height of microvilli can also be rearranged in response to cell volume change (Gorelik et al., 2003, 2004).

**Figure 2:**
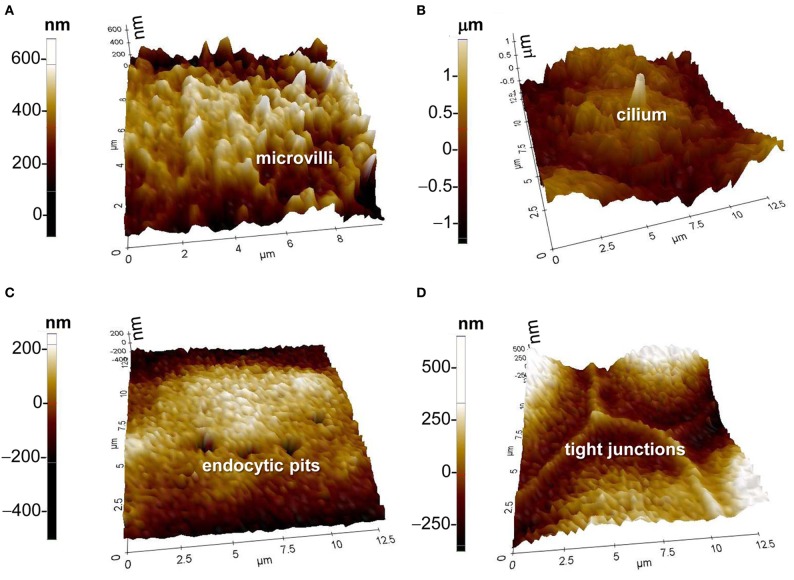
**SICM topographic images of (A) microvilli, (B) cilium, (C) endocytic pits, and (D) tight junctions.** Images were achieved with the XE-Bio SICM which was purchased from Park Systems Corporation. The cells in **(A)** through **(D)** were from mouse cortical collecting duct mpkCCDc14 cells, which were cultured as we previously described (Helms et al., 2005).

With the introduction of hopping mode, large microvilliar structures like stereocilia of hair cells were observed at high resolution (a diameter of 100 nm or less) (Novak et al., 2009). A kinocilium (true cilium) was also visualized in young postnatal auditory hair cells. SICM has also been used to investigate the dynamic changes of tight junctions. In renal epithelial cells, the morphological changes of tight junctions after hypertonic stress was visualized by SICM and used to determine the role of tight junctions in maintaining epithelial monolayer integrity (Zhang et al., 2005). Besides microvilli, cilia, and tight junctions, the endocytic pit is another specialized membrane structure which can be visualized by SICM (Shin and Gillis, 2006; Shevchuk et al., 2008a). Recent studies have shown that this powerful tool can even be used to visualize the process of clathrin-coated pit closure (Shevchuk et al., 2012). In summary, SICM is a very powerful nanotechnology to study specialized membrane structures in live cells. The use of SICM might be extended to the study of the mechanism of cilium formation or loss in the normal and polycystic kidney disease in renal epithelial cells. Recently, my laboratory has established this nanotechnology and has examined microvilli, cilia, endocytic pits, and tight junctions in mouse cortical collecting duct mpkCCD_c14_ cells (Figure 2).

### Navigate patch-clamp recordings of ion channels in specialized membrane structures

Since SICM uses glass micropipette filled with electrolyte, after achieving topographical image by scanning the cell surface the same micropipette can be used for patch-clamp recording of ion channels. In other words, SICM provides the nanoscale navigation for patch-clamp recording of single ion channel activity at defined positions or structures on the cell surface. This so-called high-resolution or “smart” patch-clamp technique was initially developed by Gu et al. (2002). They successfully mapped ion channels in cardiac myocytes: L-type Ca^2+^ channels and Cl^−^ channels are located only in the regions of T-tubule openings. Using the conventional patch-clamp technique, the maxi-anion channel was hardly caught because the “smart” patch-clamp technique showed that the channel is mainly located at the openings of T-tubules and along Z-lines in adult cardiomyocytes (Dutta et al., 2008). After detecting the membrane structures of isolated guinea-pig ventricular myocytes with a lateral resolution of 1.86 μm, a cystic fibrosis conductance regulator-like Cl^−^ channel was precisely localized in these myocytes, which are located both in *z*-grooves and in the inter-groove region, but not at the mouths of *t*-tubules (James et al., 2010). As described above, SICM can also visualize specialized membrane structures such as microvilli and tight junctions. The “smart” patch technique makes it possible to easily locate the patch pipette at a region of interest on the cell surface including microvilli and tight junctions to record the ion channel activity (Gorelik et al., 2004; Yang et al., 2010). Since atomic force microscopy showed that the epithelial sodium channel (ENaC) is predominantly located in the microvilli of distal nephron cells (Smith et al., 1997), it would be very interesting to detect how this unique localization is related to ENaC function and regulation.

### Provide a topographic background for confocal microscopy localization of protein molecules and nanoparticles

SICM can be combined with simultaneous confocal fluorescence imaging, resulting in a new technique named scanning surface confocal microscopy. This technique allows investigators to study the relationship between cell surface membrane structures and fluorescently labeled proteins or nanoparticles in both fixed and live samples. SICM can be performed at a sufficient resolution on living cells to image fixed or slowly diffusing individual protein complexes in the plasma membrane and to follow their reorganization over time. Such proteins are likely to be present on highly structured cells where specific functions are associated with particularly specialized regions or domains. The cell membrane exhibits numerous submicrometer-sized surface structures that could be topographically confused with virus particles. This problem can be solved with simultaneous confocal microscopy localization of fluorescently labeled molecules. Using this technique, previous studies showed that virus-like particles were randomly distributed across the cell membrane and had no specific interactions with specialized membrane structures such as microvilli (Shevchuk et al., 2008b). The combination of SICM with confocal microscopy also allows investigators to study nanoparticle uptake. Previous studies showed that nanoparticles with two different sizes (50 nm and 1 μm) can be internalized in equal numbers by human alveolar type-1-like cells (Kemp et al., 2008). In addition to the nanoparticles described above, this technique also can be applied to any virus or other nanoparticle such as gene delivery agents as long as they can be labeled with a fluorescent moiety. It is anticipated that this procedure will be useful in characterizing the earliest interactions of these particles with the cell and contribute to identifying novel drugs to prevent viral infection as well as in the development of gene therapy reagents. This technique should find widespread application for studying the relationship of fluorescently tagged molecules within the cell membrane.

### Monitor cell volume and movements

Regulation of cell volume is a fundamental mechanism for cellular homeostasis. Changes in cell shape, movement, and volume reflect normal cellular functions such as secretion, ion transport, and adaptation to environment. In order to study the physiology and pathology associated with the regulation of cell volume and cell membrane movement, an appropriate technique is needed, which allows quantitative and high-resolution characterization without influencing the cell functionality. SICM is one of the most advanced ways to meet these requirements. The technique can measure a wide range of volumes from 10^−19^ to 10^−9^ liter. The cell volume, as well as the cell volume of small cellular structures such as dendrites, processes, or microvilli, can be measured at the 2.5 × 10^−20^ liter resolution (Korchev et al., 2000a; Kemp et al., 2008). Membrane movements in the range of 30 min to a few hours can be quantitatively monitored with SICM at a lateral resolution of 500 nm (Happel et al., 2003; Kemp et al., 2008). Through monitoring the volume of either cells or cellular structures, SICM is a powerful tool to investigate how a functional epithelial monolayer maintains its integrity when given hypertonic stress (Zhang et al., 2005).

### Deliver mechanical or chemical stimulations

Mechanosensitive ion channels convert external mechanical force into electrical and chemical signals in cells, but their physiological function is hard to detect. SICM is a satisfactory physiological tool to stimulate these channels in living cells by means of a nanopipette. This method can be straightforwardly extended to noncontact mapping of both the mechanical properties of the cell and mechanosensitive ion channels (Sanchez et al., 2007). Sanchez et al. produced a non-contact, controlled mechanical stimulation on human and rat dorsal root ganglia sensory neurons and, simultaneously, used whole-cell patch-clamp recordings and measurements of intracellular Ca^2+^ concentration to validate the effects of mechanical stimulation on mechanosensitive ion channels. SICM can provide repetitive mechanical stimulation of a specific cellular region without introducing any damage, and serves as a stable model to study cell mechanotransduction and test novel drugs that may inhibit or modulate mechanosensitive ion channels. When the scanning is performed near the cell membrane, SICM can be used to interact with growing neurons to induce their remodeling (Pellegrino et al., 2009, 2011). Since the guidance of axon growth cones to their targets is essential for establishing neural circuits, the technique can significantly promote neuroscience research.

## Conclusions

SICM, combined with confocal microscopy and patch-clamp techniques, is a unique technique to investigate membrane topography at a nanometer scale and single protein function in specific membrane microdomains such as microvilli, cilia, endocytic pits, or tight junctions, especially in live cells. This nanotechnology will continue to generate important new insights in biological studies.

### Conflict of interest statement

The authors declare that the research was conducted in the absence of any commercial or financial relationships that could be construed as a potential conflict of interest.
